# Supplementary material for: Novel Methods of Targeting IL-1 Signalling for the Treatment of Breast Cancer Bone Metastasis
Source: Cancers (Basel). 2022 Oct 1;14(19):4816. doi: 10.3390/cancers14194816 (PMC9561984; doi:10.3390/cancers14194816)
Supplement: Supplementary file 1 [file cancers-14-04816-s001.zip › cancers-1913963-supplementary.pdf]

cancers MDPI

Supplementary Table S1

Table S1. Antibody of lymphocyte and myeloid for flow cytometry

	Antibody (Clone)	Fluorophore	Filter	Cat. (BioLegend)	Dilution
Lym-phocyte Panel	Viability	Zombie UV	UV450	423108	1:100
	CD19 (6D5)	PE	B575	115508	1:100
	CD4 (GK1.5)	PerCP/Cya-nine5.5	B695	100433	1:100
	NK1.1 (PK136)	PerCP/ Cya-nine7	B780	108713	1:100
	CD45 (30-F11)	Brilliant Vi-olet421	V450	103134	1:100
	CD3 (17A2)	Brilliant Vi-olet510	V525	100234	1:100
	CD8 (CD8a)	Alexa Fluor674	R660	100724	1:100
Myeloid panel	Viability	Zombie UV	UV450	423108	1:100
	Ly6C (HK1.4)	PE	B575	128008	1:100
	CD11c (N418)	PE-eFluro610	B610	61-0114-82 (Invitrogen)	1:100
	Ly6G (1A8)	PerCP/Cya-nine5.5	B695	127615	1:100
	F4/80 (BM8)	PE/Cya-nine7	B780	123113	1:100
	CD45 (30-F11)	Brilliant Vi-olet421	V450	103134	1:100
	MHCII (M5/114.15.2)	Brilliant Vi-olet510	V525	107636	1:100
	MRC1 (C068C2)	Alexa Fluor700	R730	141733	1:100

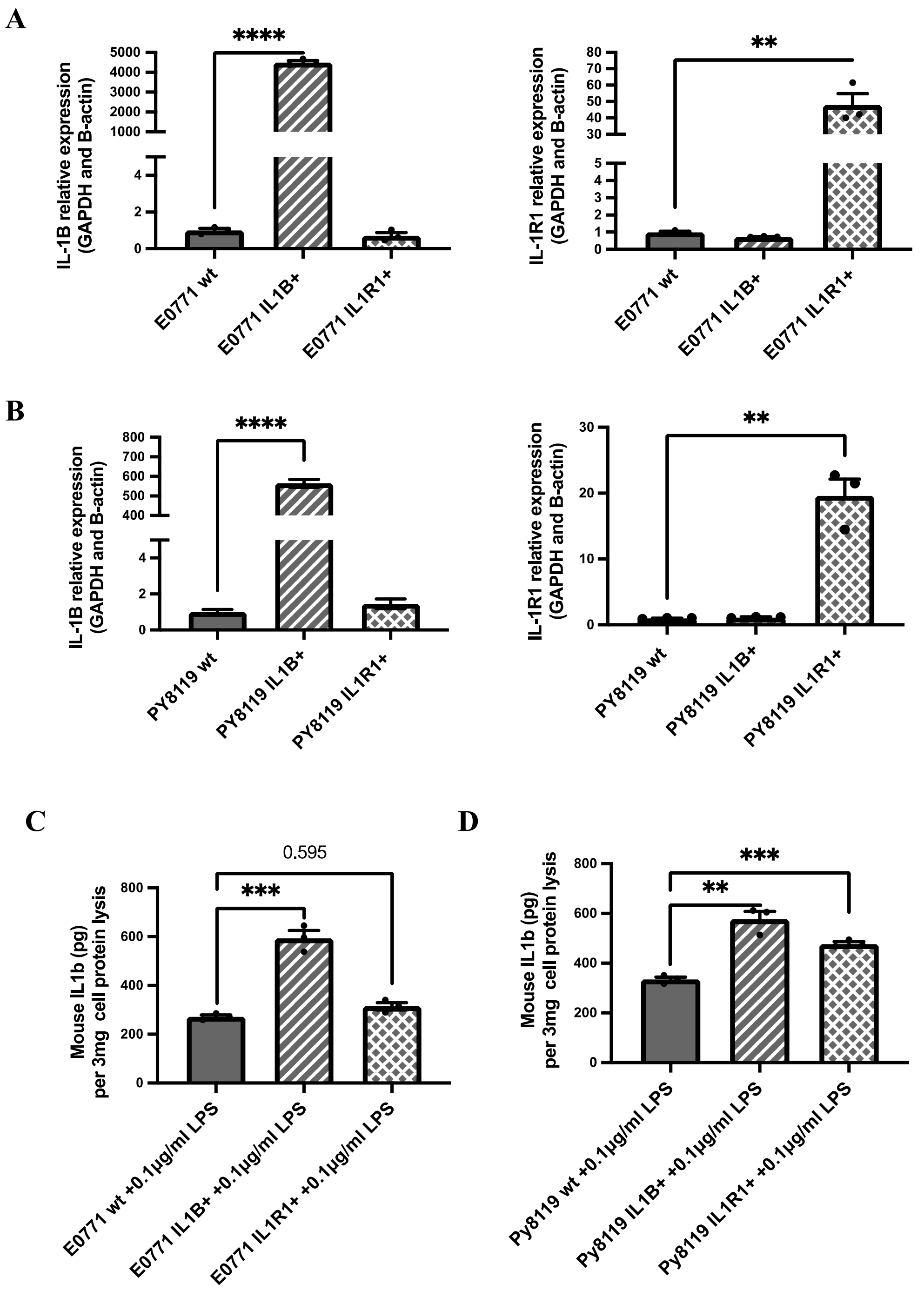

Figure S1. (A) Relative expression of IL1B and IL1R1 compared with GAPDH and B-actin in E0771 IL1B+, E0771 IL1R1+ and E0771 wild-type (wt) cell lines measured by Real-time PCR. (B) Relative expression of IL1B and IL1R1 compared with GAPDH and B-actin in Py8119 IL1B+, Py8119 IL1R1+ and Py8119 wt cell lines measured by Real-time PCR. (C) IL1β production in E0771 IL1B+, E0771 IL1R1+ and E0771 wild-type (wt) cell lines and (D) IL1β production in Py8119 IL1B+, Py8119 IL1R1+ and Py8119 wt cell lines after 24 hours LPS pre-treatment. Data are shown as mean ± SD. *$p < 0.05$, **$p < 0.01$, ***$p < 0.001$, ****$p < 0.0001$

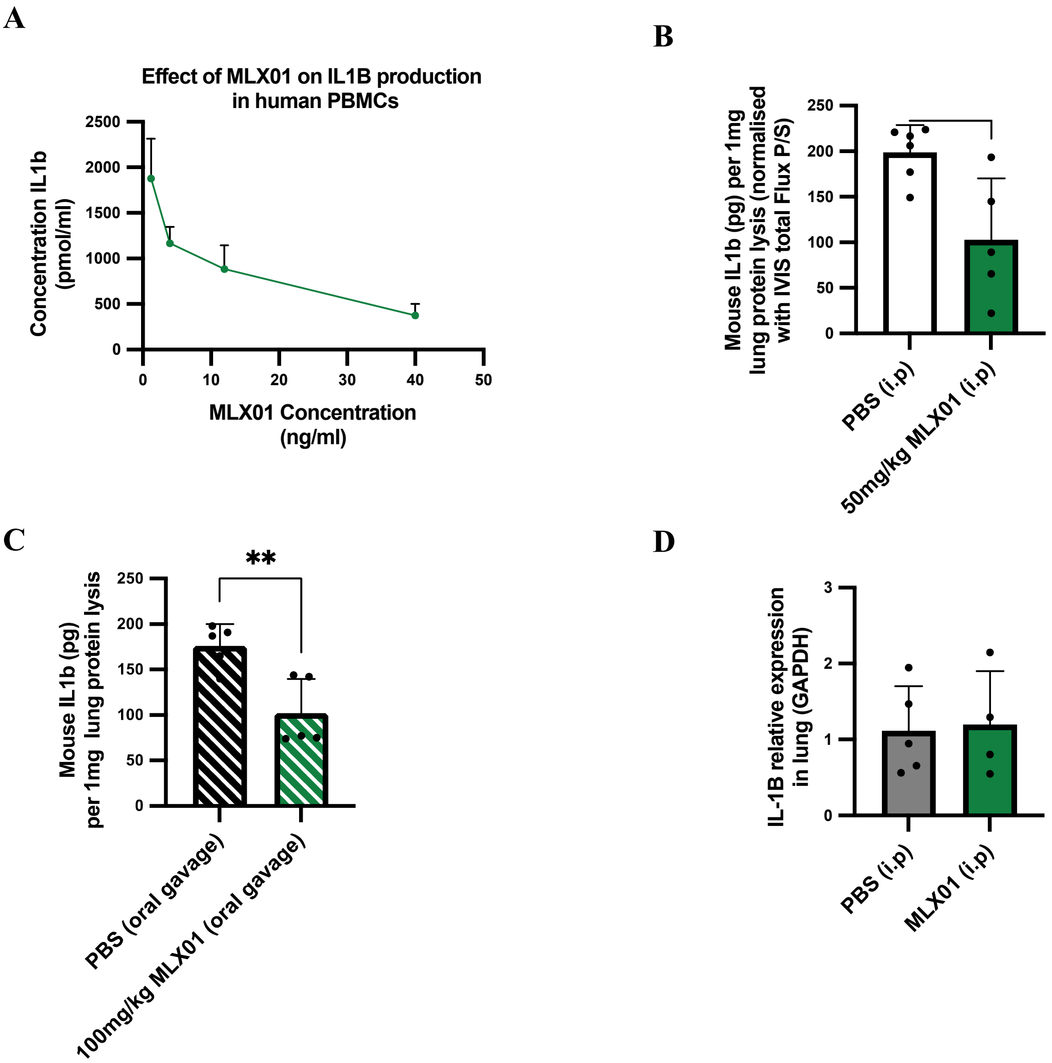

Figure S2. (A) Effect of MLX01 on IL1β production in human peripheral blood mononuclear cells (PBMCs) after 24 hours pre-treatment. $5X10^6$ PBMCs were activated with LPS ((Lipopolysaccharides from Salmonella enterica serotype typhimurium). LPS was added at a concentration of 0.1 μg per ml. before addition of MLX01. IL1β concentrations (as pmoles/ml) were measured via cytometric bead array (BD Accuri C6 Plus Flow Cytometer using BD CBA Flex sets). (B) IL1β concentration in protein lysates from lungs of mice injected with 50mg/kg MLX01 i.p. (From Figure 4A), and it was normalised to total Flux photons/second (Figure 4G). (C) IL1β concentration in protein lysates from lungs of mice treated with 100mg/kg MLX01 by oral gavage (From Figure S6A). (D) Relative expression of IL1B compared with GAPDH in lungs following administration of PBS and 50mg/kg MLX01 via i.p injection. Data are shown as mean + SD. * $p < 0.05$, ** $p < 0.01$

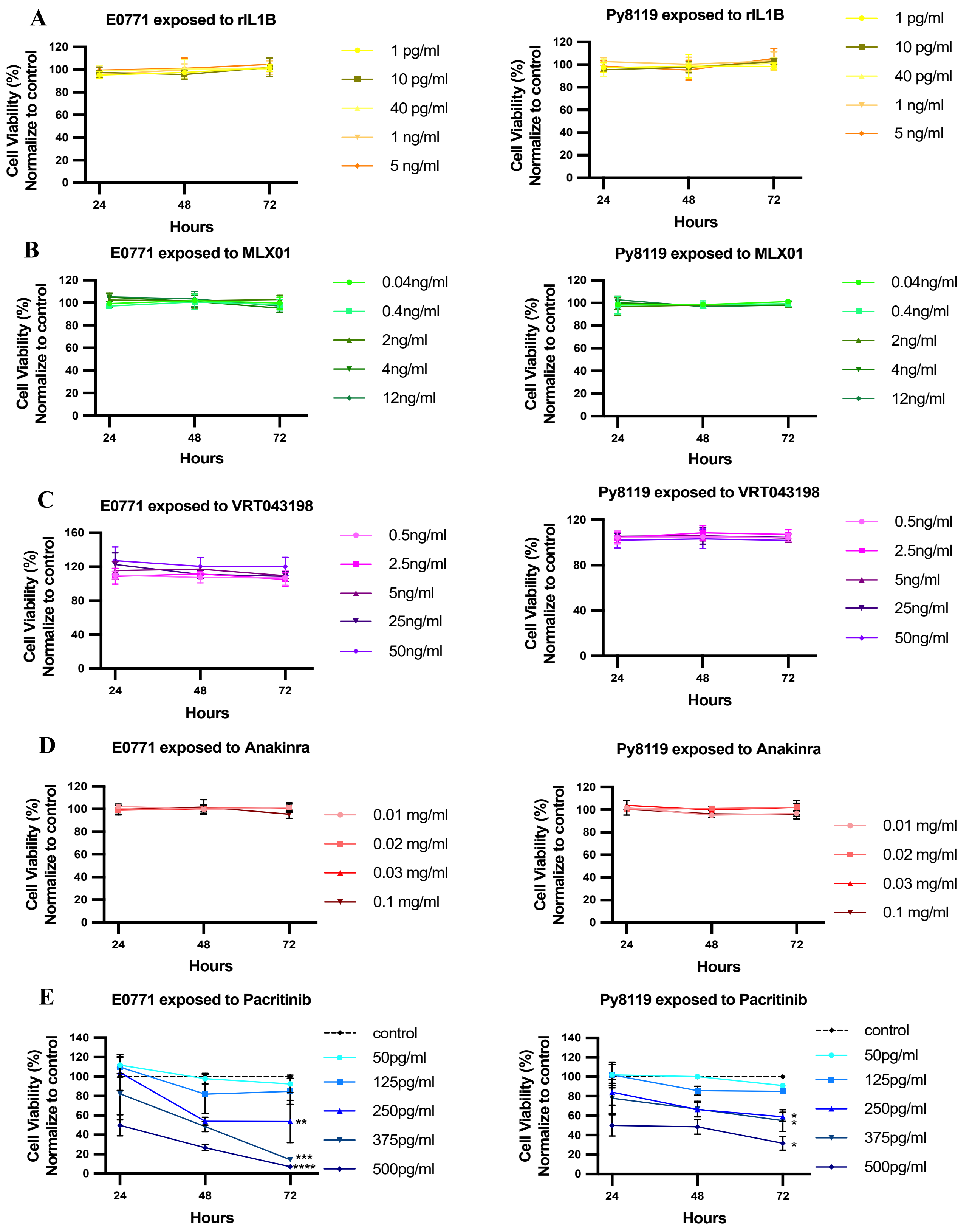

Figure S3. The relative viability of E0771 and Py8119 cells exposed to (A) Recombinant IL1β, (B) IL1β inhibitor MLX01, (C) Caspase-1 inhibitor VRT043198, (D) IL1R antagonist Anakinra and (E) IRAK1 inhibitor Pacritinib. Data are shown as mean ± SD. * $p < 0.05$, ** $p < 0.01$, *** $p < 0.001$, **** $p < 0.0001$

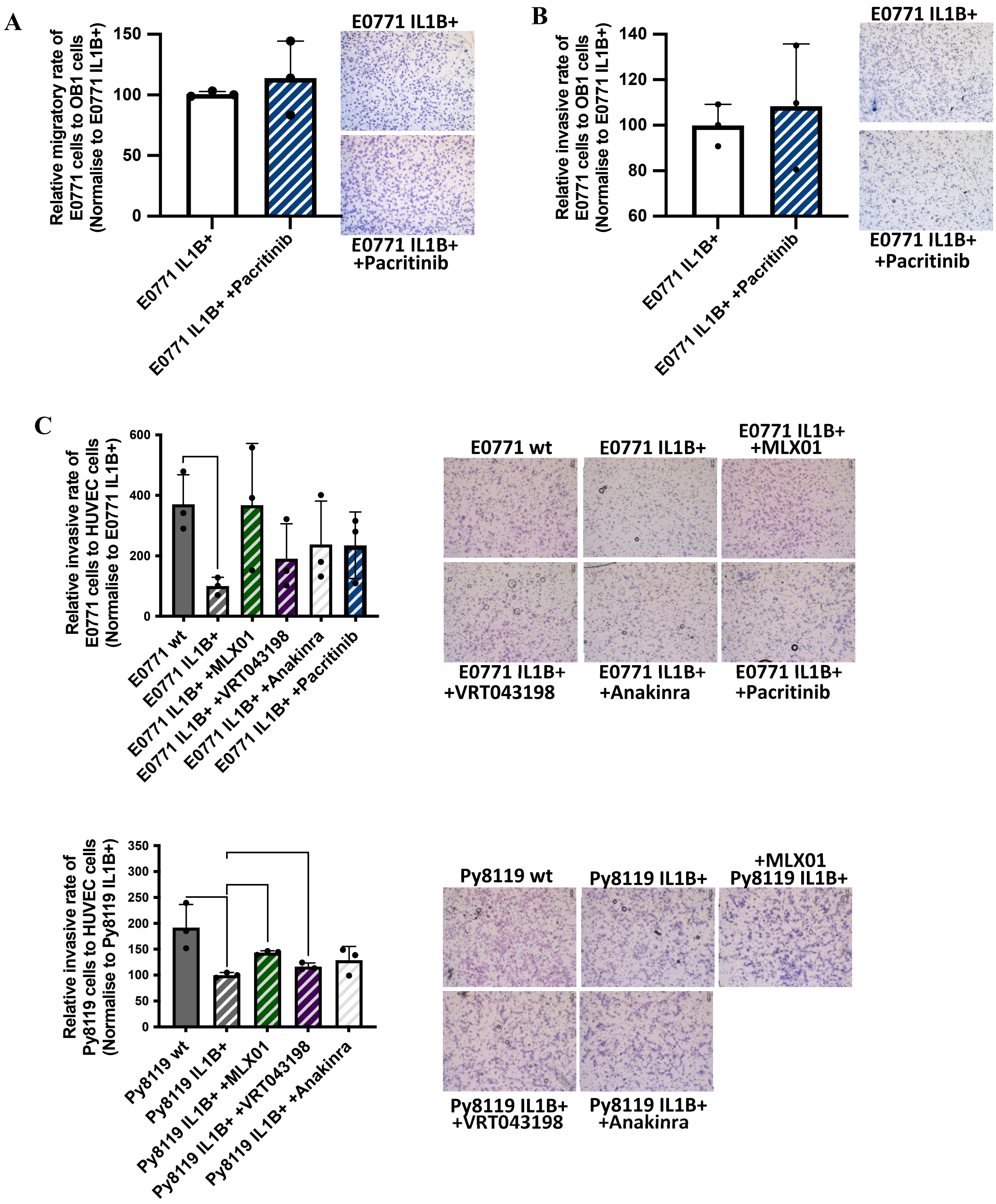

Figure S4. Inhibition of IRAK1 by Pacritinib had no significant effect on migration and invasion of E0771 IL1B+ cells towards pre-osteoblast (OB1 cells) data are shown in (A,B). (C) The effects of alteration of IL1β signalling of E0771 and Py8119 on migration and invasion towards umbilical vein endothelial cells for 24 hours (HUVECs cells). Data are shown as mean ± SD. *p < 0.05, **p < 0.01, ***p < 0.001

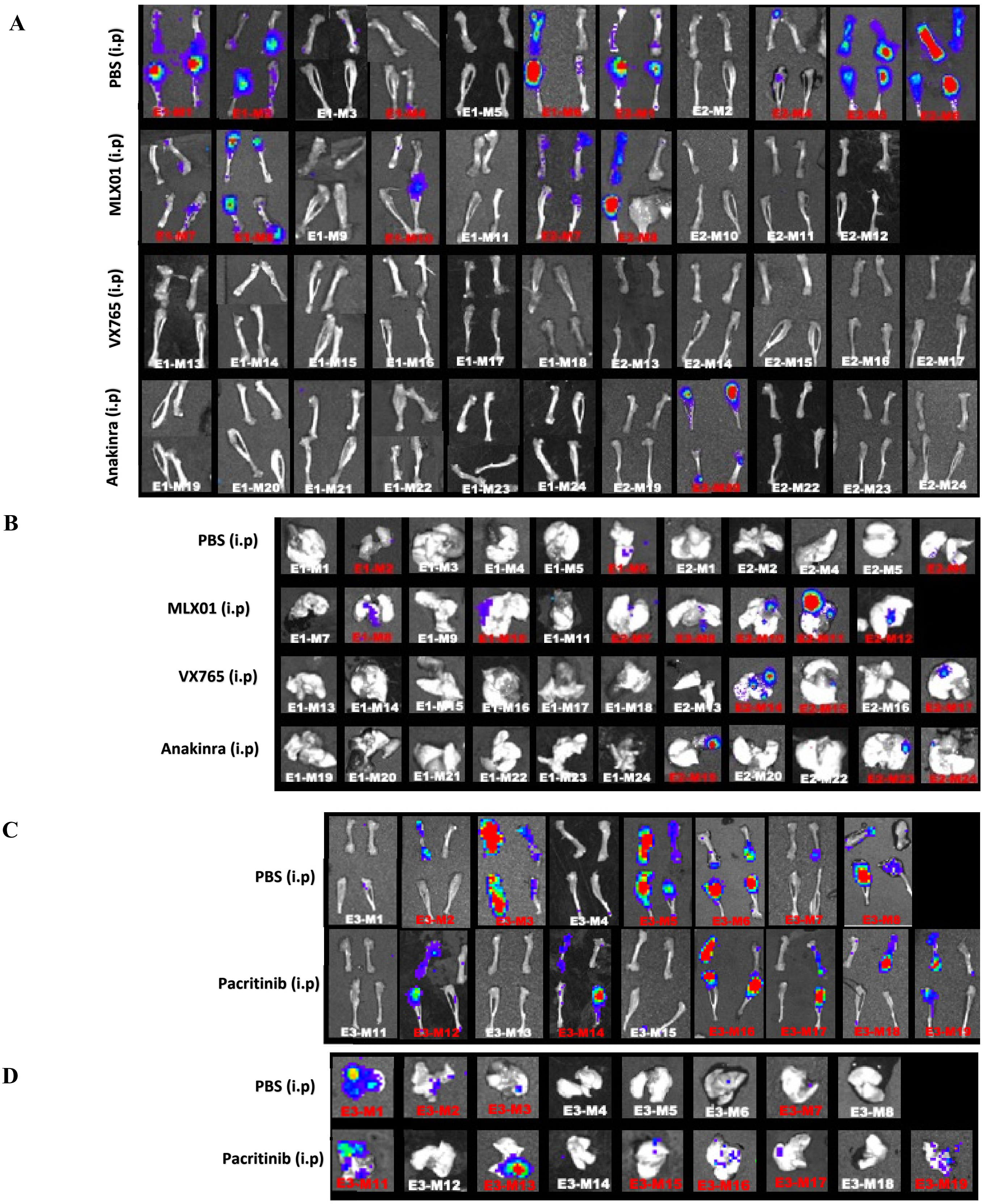

Figure S5. (A) Images of IVIS imaging of dissected femur/tibia in ex vivo from study Figure 3A. (B) Images of IVIS imaging of dissected lung in ex vivo from study Figure 3A. (C) Images of IVIS imaging of dissected femur/tibia in ex vivo from study Supplementary Figure 3H. (D) Images of IVIS imaging of dissected lung in ex vivo from study Supplementary Figure 3H.

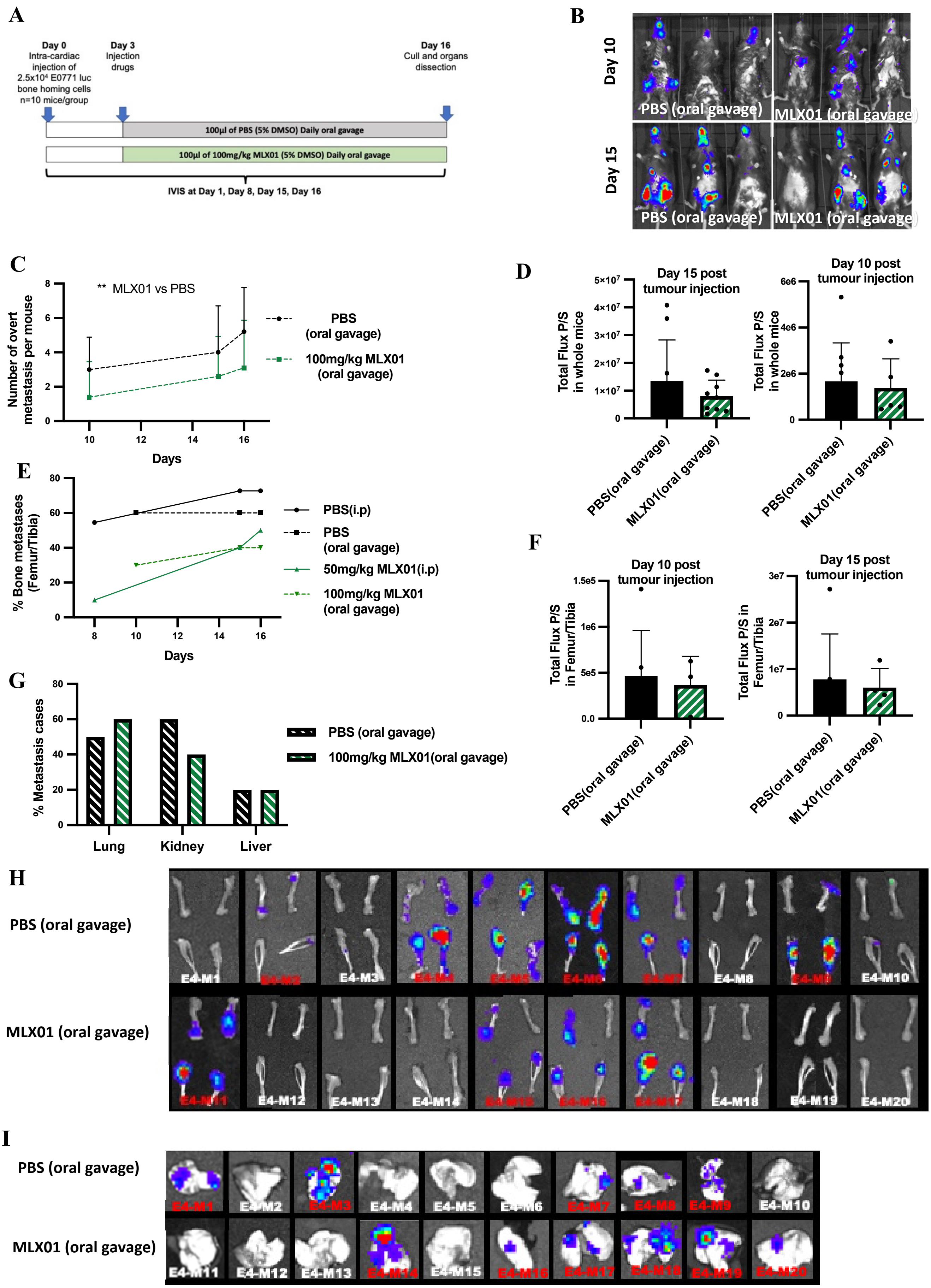
A
Day 0
Intra-cardiac injection of 2.5x10⁴ E0771 luc bone homing cells
n=10 mice/group
Day 3
Injection drugs
Day 16
Cull and organs dissection
100µl of PBS (5% DMSO) Daily oral gavage
100µl of 100mg/kg MLX01 (5% DMSO) Daily oral gavage
IVIS at Day 1, Day 8, Day 15, Day 16
B
Day 10
Day 15
PBS (oral gavage)
MLX01 (oral gavage)
C
** MLX01 vs PBS
Number of overt metastasis per mouse
Days
PBS (oral gavage)
100mg/kg MLX01 (oral gavage)
D
Day 15 post tumour injection
Day 10 post tumour injection
Total Flux P/S in whole mice
PBS(oral gavage)
MLX01(oral gavage)
E
% Bone metastases (Femur/Tibia)
Days
PBS(i.p)
PBS (oral gavage)
50mg/kg MLX01(i.p)
100mg/kg MLX01 (oral gavage)
F
Day 10 post tumour injection
Day 15 post tumour injection
Total Flux P/S in Femur/Tibia
PBS(oral gavage)
MLX01(oral gavage)
G
% Metastasis cases
Lung
Kidney
Liver
PBS (oral gavage)
100mg/kg MLX01(oral gavage)
H
PBS (oral gavage)
MLX01 (oral gavage)
E4-M1 E4-M2 E4-M3 E4-M4 E4-M5 E4-M6 E4-M7 E4-M8 E4-M9 E4-M10
E4-M11 E4-M12 E4-M13 E4-M14 E4-M15 E4-M16 E4-M17 E4-M18 E4-M19 E4-M20
I
PBS (oral gavage)
MLX01 (oral gavage)
E4-M1 E4-M2 E4-M3 E4-M4 E4-M5 E4-M6 E4-M7 E4-M8 E4-M9 E4-M10
E4-M11 E4-M12 E4-M13 E4-M14 E4-M15 E4-M16 E4-M17 E4-M18 E4-M19 E4-M20

Figure S6. Administration of 100mg/kg MLX01 by oral gavage delayed onset of breast cancer bone metastasis. (A) Protocol outline for intra-cardiac injection of E0771 cells and administration of 100mg/kg MLX01 (oral gavage) in mice. (B) Representative images of IVIS imaging of the whole body of mice at Day 10 and Day 15 post E0771 cells injection. (C) Numbers of overt metastasis tumours per mouse post 10, 15 and 16 days E0771 cells injection following daily administration of PBS (control), or 100mg/kg MLX01 by oral gavage. (D) Total luciferase expressing E0771 tumour cells (photons per second (P/S)) of whole mice post 10, 15 days E0771 cells injection. E) Numbers of mice that developed bone metastases 10, 15 and 16 days after E0771 cells had been disseminated in bone following daily administration of PBS (control) or MLX01. (F) Size of tumours that grew in bone as assessed by photons per second (P/S) in luciferase expressing E0771 tumour cells. (G) Shows effects of PBS (control) or 100mg/kg MLX01 by oral gavage on metastatic outgrowth of tumours following dissemination into the lungs, kidneys and livers. (H) Images of IVIS imaging of dissected femur/tibia in ex vivo. (I) Images of IVIS imaging of dissected lung in ex vivo. The graphs represent mean + SD, *$p < 0.05$, **$p < 0.01$

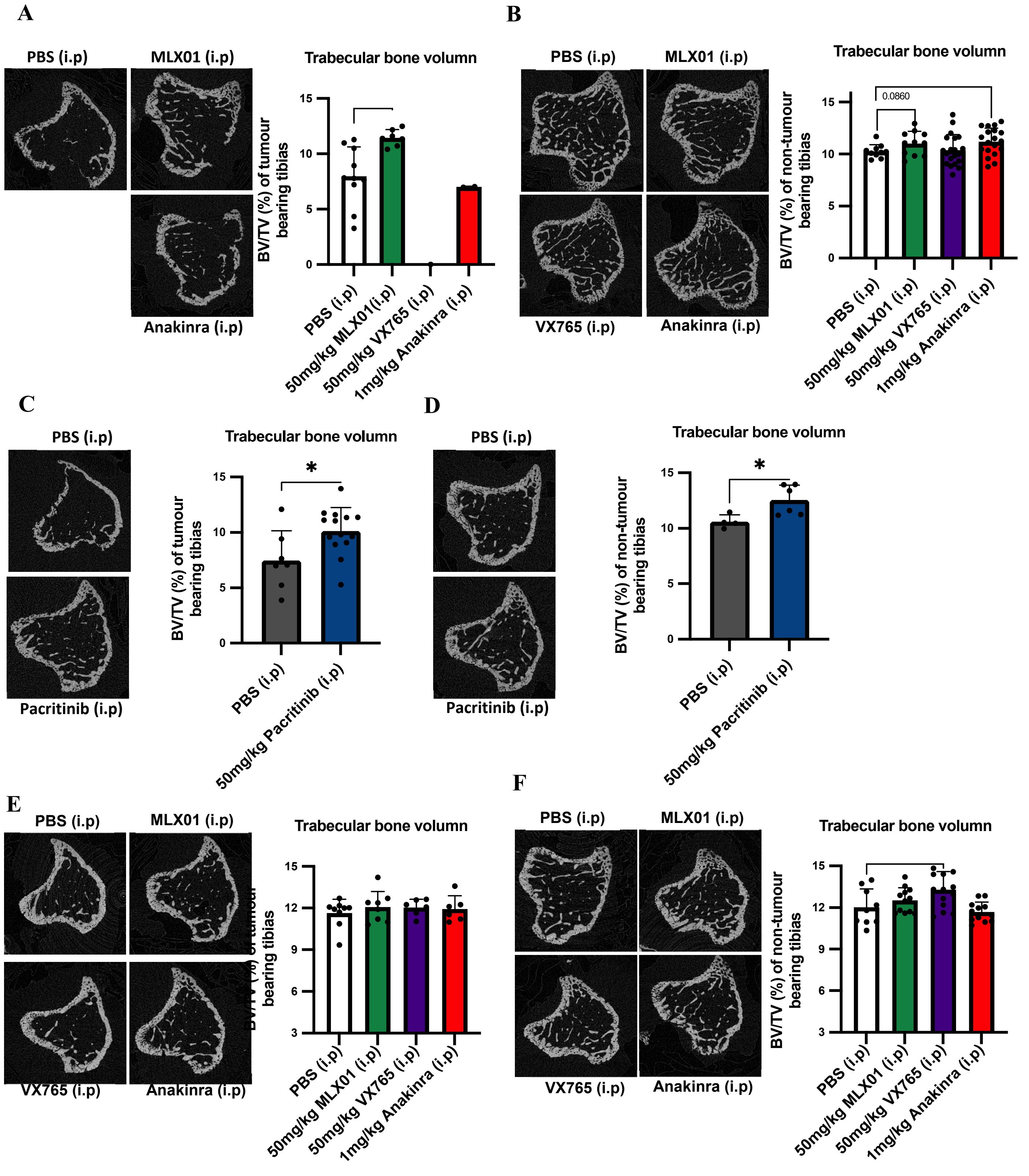

Figure S7. (A,B) BV/TV % of tumour-bearing or non-tumour-bearing tibias in Figure 5A. (C,D) BV/TV % of tumour-bearing or non-tumour-bearing tibias in Figure 5B. (E,F) BV/TV % of tumour-bearing or non-tumour-bearing tibias in Figure 5C. The graphs represent mean + SD, $^*p < 0.05$

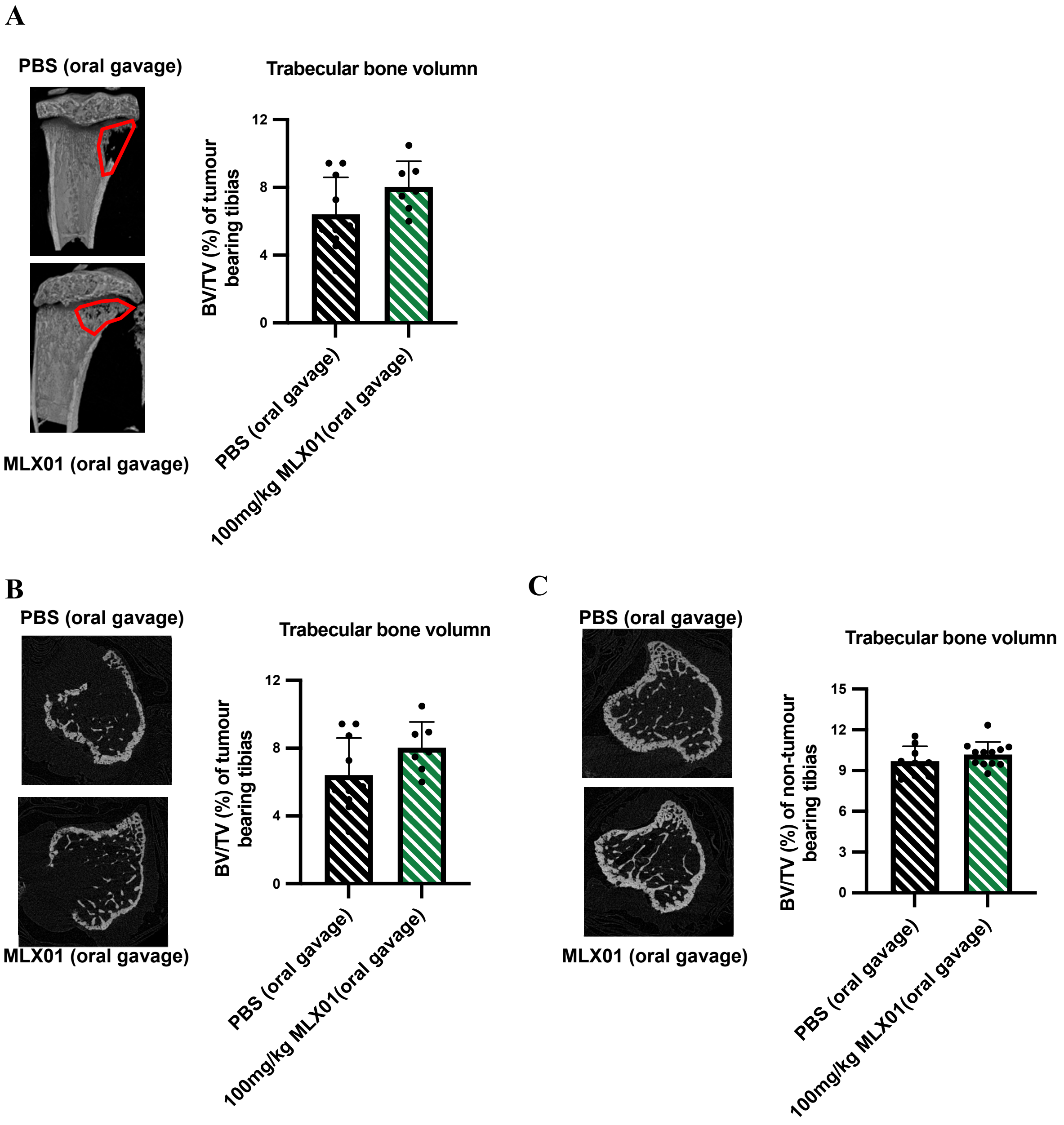

Figure S8. Effect of administration of MLX01 oral gavage on trabecular bone volume BV/TV % are shown in (A). and BV/TV % of tumour-bearing or non-tumour-bearing tibias in (B,C). The graphs represent mean + SD, *$p < 0.05$

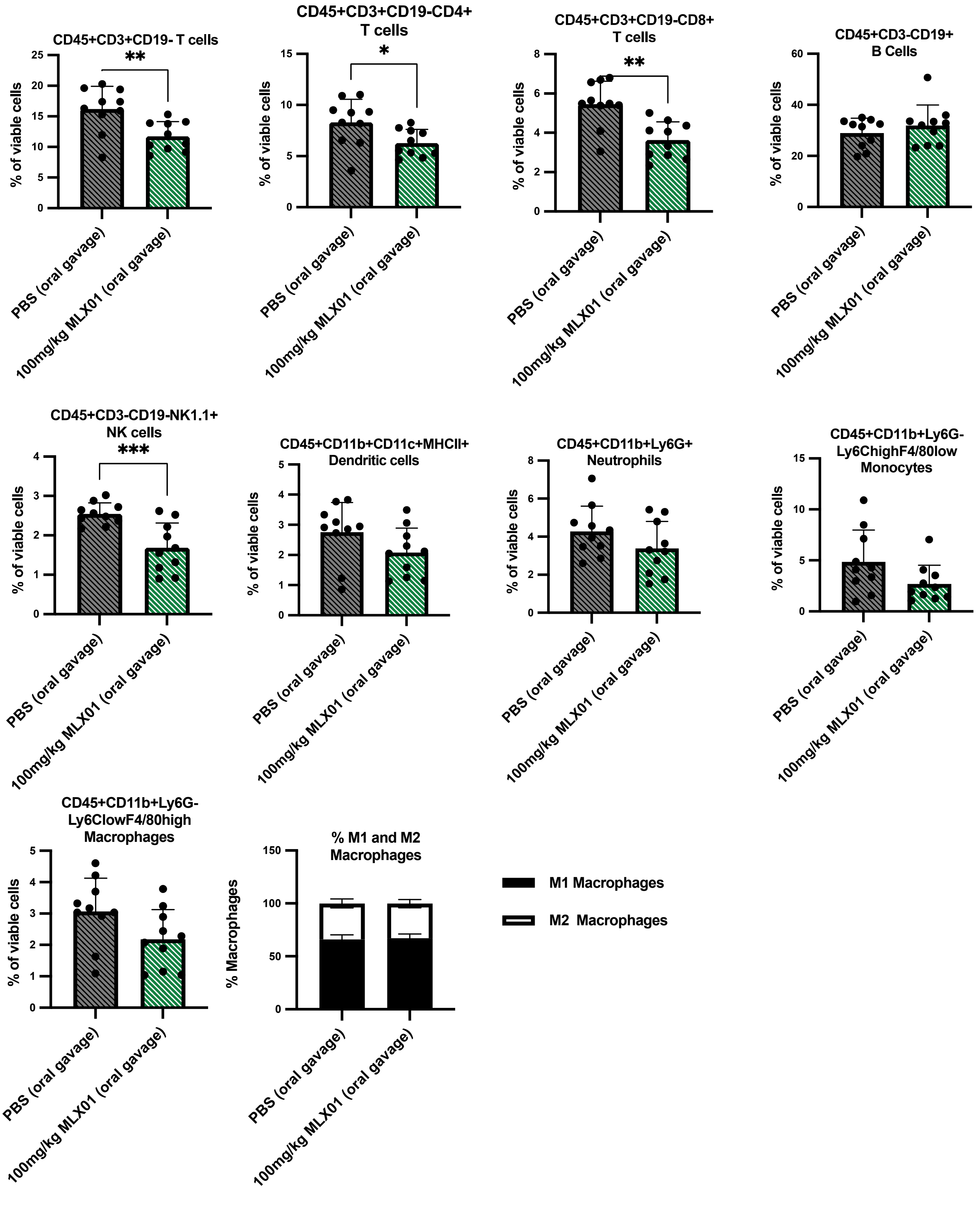

Figure S9. Effects of MLX01 on systemic immune cell populations The graphs rep resent mean + SD, * p < 0.05, ** p < 0.01

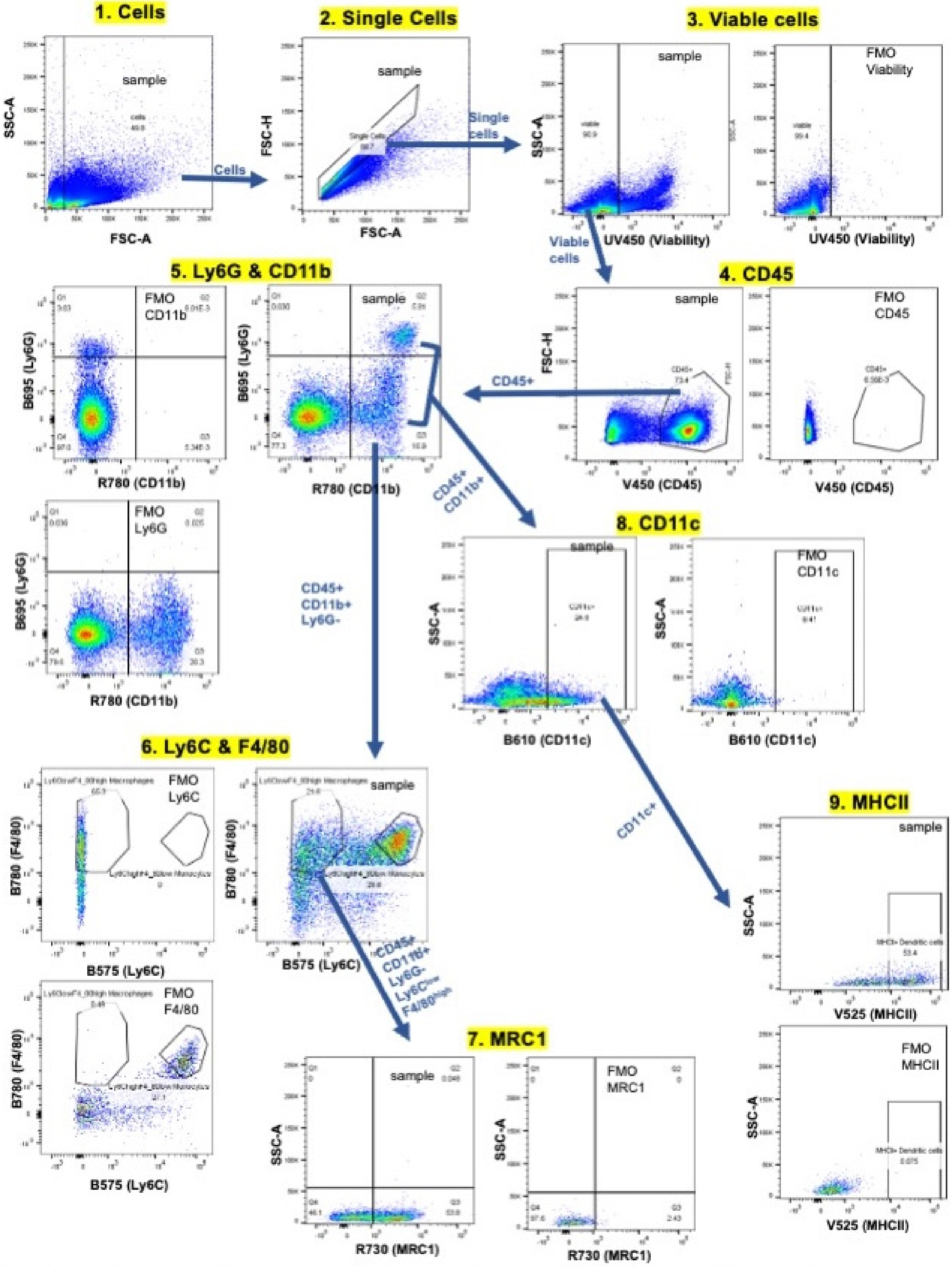

NB Gating is shown as the fluorescence minus one (FMO) control and a representative sample

Figure S10. Myeloid cells gating Neutrophils (CD45+CD11b+Ly6G+) is classified by step 5. Macrophage (CD45+CD11b+Ly6G-Ly6ClowF4/80high) and Monocytes (CD45+CD11b+Ly6G-Ly6ChighF4/80low) are classified by step 6. M1 Macrophage (CD45+CD11b+Ly6G-Ly6ClowF4/80highMRC1-) and M2 Macrophage (CD45+CD11b+Ly6G-Ly6ClowF4/80highMRC1+) are classified by step 7. Dendritic cells (CD45+CD11b+CD11c+MHCII+) is classified by step 9.

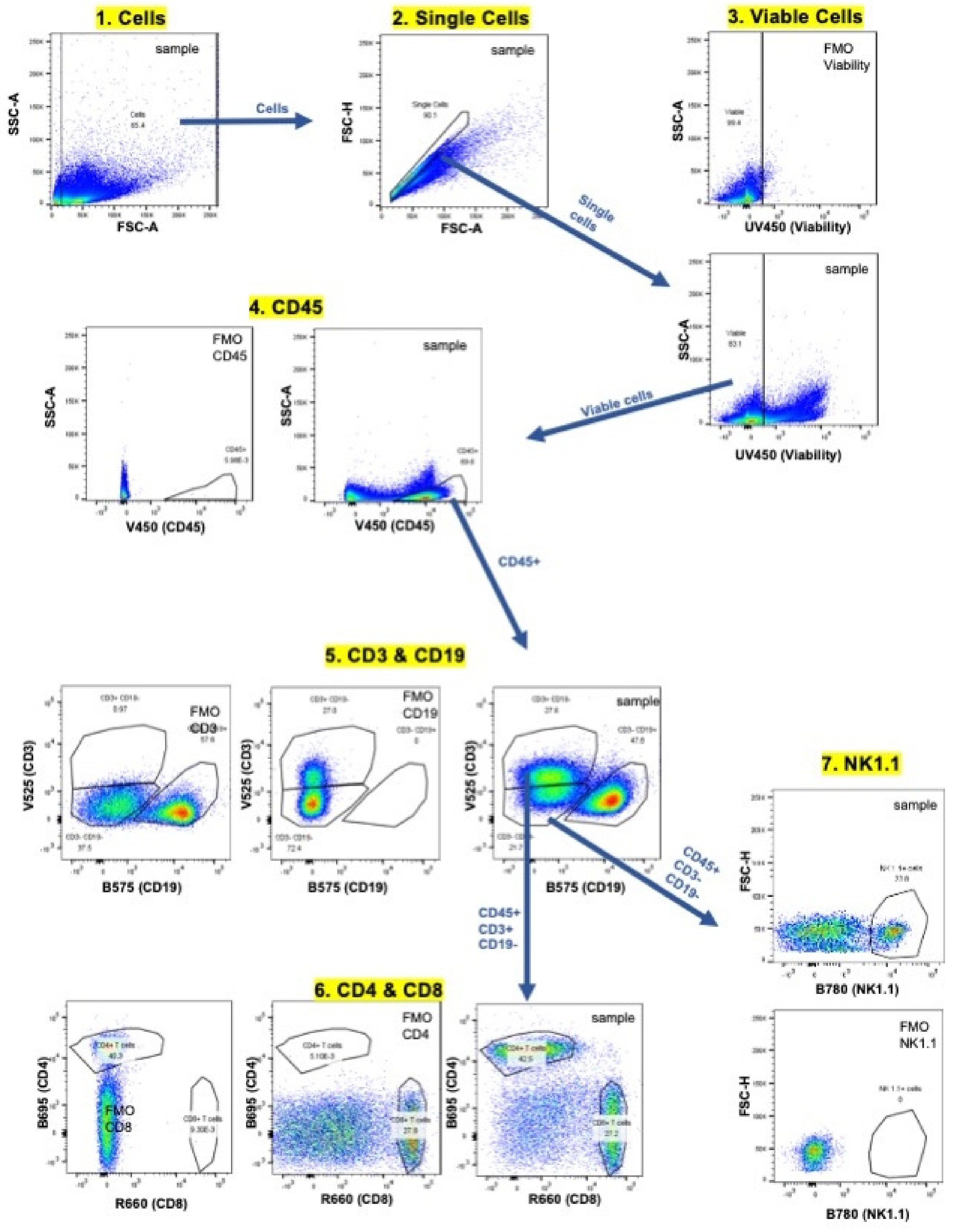

NB Gating is shown as the fluorescence minus one (FMO) control and a representative sample

Figure S11. Lymphocyte cells gating T cells (CD45+CD3+CD19-) and B cells (CD45+CD3-CD19+) are classified by step 5. CD4 T cells and CD8 cells are classified by step 6. Natural killer cells (CD45+CD19-NK1.1+) is classified by step 7.
